# Scalable Electrophysiology of Millimeter-Scale Animals with Electrode Devices

**DOI:** 10.34133/bmef.0034

**Published:** 2023-12-07

**Authors:** Kairu Dong, Wen-Che Liu, Yuyan Su, Yidan Lyu, Hao Huang, Nenggan Zheng, John A. Rogers, Kewang Nan

**Affiliations:** ^1^ College of Pharmaceutical Sciences, Zhejiang University, Hangzhou 310058, China.; ^2^ National Key Laboratory of Advanced Drug Delivery and Release Systems, Zhejiang University, Hangzhou 310058, China.; ^3^ College of Biomedical Engineering & Instrument Science, Zhejiang University, Hangzhou, 310027, China.; ^4^ Department of Gastroenterology, Brigham and Women’s Hospital, Harvard Medical School, Boston, MA 02115, USA.; ^5^ College of Chemical and Biological Engineering, Zhejiang University, Hangzhou 310058, China.; ^6^ Qiushi Academy for Advanced Studies, Zhejiang University, Hangzhou 310027, China.; ^7^ College of Computer Science and Technology, Zhejiang University, Hangzhou 310027, China.; ^8^ State Key Lab of Brain-Machine Intelligence, Zhejiang University, Hangzhou 310058, China.; ^9^ CCAI by MOE and Zhejiang Provincial Government (ZJU), Hangzhou 310027, China.; ^10^ Querrey Simpson Institute for Bioelectronics, Northwestern University, Evanston, IL 60208, USA.; ^11^ Department of Biomedical Engineering, Northwestern University, Evanston, IL 60208, USA.; ^12^ Department of Materials Science and Engineering, Northwestern University, Evanston, IL 60208, USA.; ^13^ Department of Mechanical Engineering, Northwestern University, Evanston, IL 60208, USA.; ^14^ Jinhua Institute of Zhejiang University, Jinhua 321299, China.

## Abstract

Millimeter-scale animals such as *Caenorhabditis elegans*, *Drosophila* larvae, zebrafish, and bees serve as powerful model organisms in the fields of neurobiology and neuroethology. Various methods exist for recording large-scale electrophysiological signals from these animals. Existing approaches often lack, however, real-time, uninterrupted investigations due to their rigid constructs, geometric constraints, and mechanical mismatch in integration with soft organisms. The recent research establishes the foundations for 3-dimensional flexible bioelectronic interfaces that incorporate microfabricated components and nanoelectronic function with adjustable mechanical properties and multidimensional variability, offering unique capabilities for chronic, stable interrogation and stimulation of millimeter-scale animals and miniature tissue constructs. This review summarizes the most advanced technologies for electrophysiological studies, based on methods of 3-dimensional flexible bioelectronics. A concluding section addresses the challenges of these devices in achieving freestanding, robust, and multifunctional biointerfaces.

## Introduction

Millimeter-scale animals in this review refer to model animals that are smaller than model rodents and mammals. Examples include *Caenorhabditis elegans*, *Drosophila* larvae, zebrafish, bees, and *Drosophila*. Electrical measurements from these species have the potential to serve an essential role in studies of fundamental correlations between the nervous system and behaviors [1–3] and in uses for disease diagnosis and treatment [4]. Such animals have body sizes in the range of a few millimeters and body tissues with soft mechanical properties. These features demand exceptionally small, flexible device configurations for conformable 3-dimensional (3D) integration. Table 1 summarizes the physiological characteristics of several of these millimeter-scale animals. The associated unique device challenges dictate unusual design strategies and fabrication methods in 3D electronics, distinct from those that can be applied to model rodents and other mammals [5–7].

**Table 1.** Physiological characteristics of millimeter-scale animals

Millimeter-scale animals	Life span (d)	Transparency	Length (mm)	Weight (mg)	Number of neurons	Throughout (animals/d)
*C. elegans*	~14	Transparent	<0.2	<0.002	<10^3^	20–1,000
*Drosophila* larvae	~3	Transparent	3–5	~3	<10^4^	Dozens
Zebrafish	>365	Semitransparent	<40	<300	~10^5^	Dozens
Bees	>42	Nontransparent	<25	<350	~10^6^	~10
*Drosophila*	<50	Nontransparent	2.5–3	0.8–1.2	~10^5^	~10

Compared with bioelectronics for these cases, the development of corresponding systems for millimeter-scale animals is still in its infancy. The small sizes of the interfaces and the large deformations in free-moving millimeter-scale animals require fundamental advances both in the structures and in the materials for the electronics. Currently, the most common electrode interfaces for millimeter-scale animals involve patch clamping, microelectrode arrays (MEAs), and microwires. For patch clamping, the tested animals must be anesthetized or tightly constrained in a manner that limits the scope of activities and the diversity of behaviors that can be investigated [8,9]. The low throughput associated with these methods and the required cumbersome manual operations hinder the scalability of chronic electrophysiological recordings [10]. Furthermore, the invasiveness of probing electrodes may influence normal electrophysiological activities. MEAs are relatively scalable and inexpensive for large-area recording compared with patch-clamp techniques [11,12]. These devices are also less invasive than conventional methods, and they do not require animal dissections, but they fail to capture of 3D, out-of-the-plane signals, limiting the spatial resolution and leading to the loss of critical information during animal movements.

Soft, freestanding bioelectronics designed for small vulnerable organoids show great potential for scalable electrophysiological monitoring of millimeter-scale animals. A set of emerging bioelectronic technologies exploit 3D, flexible, and freestanding layouts enabled by advanced micro- and nanoscale fabrication techniques [13–15]. On the one hand, the density of electrodes integrated in these systems can match that of conventional MEAs, with the potential to monitor large groups of cellular tissues [16,17]. These electrodes distribute over a network of wires, which are sufficiently flexible given their minimum widths of 10 μm [15] and bending stiffnesses around 0.1 nN·m [18]. On the other hand, stretchable structures and materials can further improve the mechanical biocompatibility [13,19,20], enabling long-term stable recording and stimulation. Cyborg organoids, for example, use stretchable mesh electronics delivered to the surfaces of growing stem cells that begin in 2D layered formats and then merge into 3D cellular structures by organogenesis [18,19,21]. Results show that these flexible meshed electronics offer improved 3D monitoring capabilities and biocompatibility for studies of cellular tissues [6,15].

This review explores the development of 3D flexible electronics for electrophysiological recordings from millimeter-scale animals. Unlike neural electrode arrays targeting rodents and mammals, bioelectronics for millimeter-scale animals must be softer, smaller in size, and with greater stretchability, outside of the scope of previous reviews of 3D flexible bioelectronics [5,6,22]. This review focuses on current electrode devices for small-scale in vivo electrophysiology and discusses critical areas for improvement. The framework casts 3D flexible electronics into 4 categories in terms of implementation methods, including ultrathin, stretchable, stress-driven assembly and biohybrid electronics, and analyzes the potential of each to be applied to millimeter-scale animals. A final section presents the challenges and next steps in unleashing the full potential of 3D flexible bioelectronics for studies of millimeter-scale animals.

## Existing Electrophysiological Technologies for Millimeter-Scale Animals

Millimeter-scale animals discussed in this work correspond to model animals that have smaller body sizes and less anatomical complexity than mammalian models [23]. The advantages of using zebrafish and *Drosophila* larvae models, for example, include their physiological resemblance to mammals [24], ease of genetic manipulations [25], sensitivity to pharmacological and genetic factors [26], robust behavior, and cost-effectiveness [27]. The genomes of the *C. elegans* are editable [28], and they are transparent with a fixed somatic cell lineage [29], making them ideal for studies on the molecular, genetic, and cellular basis of the aging process.

**Fig. 1.**
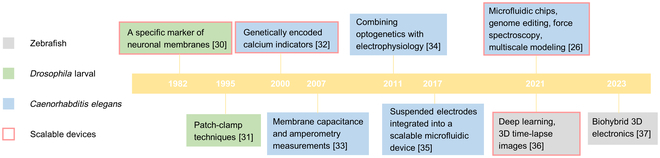
Highlights of the techniques in electrophysiology recording of millimeter-scale model animals.

This section introduces the most popular methods for electrophysiological studies of such types of millimeter-scale animals, including *C. elegans*, zebrafish, and *Drosophila* larvae, which are well-established subjects of research in neurobiology and neuroethology. A timeline that summarizes key milestones in the development of electrophysiological techniques for these applications appears in Fig. 1 [26,30–37], where the scalable technologies are highlighted with circles. These scalable technologies in terms of spatial coverage and multiple independent channels have the potential to yield insights into the signaling of the whole nervous systems of these organisms, which is currently unavailable in larger mammalian model animals.

As summarized in Table 2, technologies for recording local-field potentials or cellular-resolution activities of these millimeter-scale animals exist largely in 3 experimental regimes: patch clamp techniques that create high seal resistances through suction [38,39], MEAs that place externally near the nervous system of the animal [11], and microwires that reveal real-time aspects of neural activity [40].

**Table 2.** Existing technologies for electrophysiological recordings in millimeter-scale animal

Electrophysiological recording technology	Functionality	Performance/advantage	Limitation	References
Patch clamping	Recording	Real-time recording	Low throughput	[45,46]
		High-precision	Short-term recording	
Microelectrode arrays	Recording	Long-term recording	2D recording	[35,57]
	Electrical stimulation	High-throughput	Rigid	
		Free of invasiveness	Motion restriction	
Microwires	Recording	Real-time recording	Low throughput	[58,60,61,184]
	Electrical stimulation	Simple to prepare	Low biocompatibility	

### Patch-clamp technique

The patch-clamp technique represents one of the most widely used approaches to investigating neural excitability, which dates back to voltage-clamp measurements of the transmembrane current of neuronal fibers [41]. This technique establishes direct contact with the intracellular environment via the penetration of a glass micropipette and the formation of a high seal resistance that minimizes extracellular potential perturbation, allowing for high-precision measurements of the cell membrane potential. The interface between the electrode and cell membrane is critical, as it reduces the seal conductance and improves the measurement precision with reduced invasiveness [10].

For millimeter-scale model animals, e.g., zebrafish and *C. elegans*, patch-clamp techniques can be used for high-precision measurements, including the processes of membrane depolarizations and interchannel protein–protein interactions. In combination with intracellular Ca^2+^ recordings by loading cells with the Ca^2+^-sensitive dye Fluo-4, Schredelseker et al. [42] use the whole-cell patch clamping to analyze the L-type Ca^2+^ currents and gating currents of myotubes. Freshly dissociated skeletal muscle myotubes from zebrafish larvae were used to record the Ca^2+^ currents in the whole-cell patch-clamping experiment. They identified 2 distinct isoforms of the pore-forming subunit in zebrafish. These findings can serve as the starting point for a diverse range of further studies in the fields of skeletal muscle physiology, biophysics of ion channel pores, and evolutionary biology of skeletal muscle function. Combining in situ whole-cell patch clamping and quantitative locomotion analyses, Gao et al. [43] revealed the molecular mechanisms that underlie persistent activity. Before patch clamping, the *C. elegans* was immobilized on silicone-elastomer-coated glass coverslips by adhesives and for subsequent dissection. Results showed that the *C. elegans* neuromuscular system exhibits persistent rhythmic activity that contributes to the sustainability of basal locomotion. In another example, Cellot et al. [44] used patch clamping on early-stage zebrafish to investigate whether interference of the nanomaterial with neurotransmission may have a downstream outcome in the modulation of behavior. For larger millimeter-scale model animals, e.g., *Drosophila*, patch clamping can also play an important role in intracellular recordings. For example, patch clamping was used to identify 2 cell types that conjunctively encode the translational velocity and heading as *Drosophila* walks [45]. Similarly, Okubo et al. [46] revealed a neural network for wind-guided compass navigation with patch clamping.

Other advanced high-throughput patch-clamp technologies include automated approaches that are now available for ion channel research, reducing the need for advanced experimental expertise [47]. For example, Seibertz et al. [48] used automated patch-clamp methods to acquire a broad range of detailed electrophysiological parameters, including action potential, L-type calcium current and basal inward rectifier current. Continued work on these techniques aims to reduce interdiffusion, tissue damage, for long-term recordings [10].

### Microelectrode arrays

MEAs, much like those used to record electrophysiological signals from in vitro cell cultures [21,49], can be applied to electrophysiology of behaving millimeter-scale animals. For example, patterned MEAs can be integrated with microfluidic channels tailored to trap animals such as zebrafish [12]. These devices record relatively nonspecific local-field potentials such as electroencephalogram signals in zebrafish [11,50] but are scalable and noninvasive. Nevertheless, recording cellular electrophysiological activity deep within millimeter-scale animals, such as those beneath the muscle wall, can be difficult [35].

Advanced techniques in nano- and microfabrication serve as the basis for bioelectronics with needle-like electrodes for cellular-level interfaces deep inside the body of the small animal [51]. For example, the Michigan probe, silicon-based MEAs for neural recording, offers sufficient signal-to-noise ratio for single-neuron-resolved action potential discrimination [52]. In addition to neural interfaces, bioelectronics wrapped around millimeter-scale animals can also yield cellular-level recordings. Needle-like nanoelectrodes, some with sizes smaller than the diameter of a single cell, are available as interfaces with *C. elegans* for bioelectric recordings through muscles [35]. The structural characteristics of these needle-like nanoelectrodes are also applicable to recordings from neurons and cardiomyocytes [16,53].

Other kinds of MEAs rely on nanoscale and suspended needle-like electrodes that press [35,50] or drive [16,54] inside the animal body. Compared with patch-clamp techniques or 2D MEAs, these platforms serve as interfaces with millimeter-scale animals for bioelectric recordings at a local scale. In hybrid nanoelectronic/fluidic devices, nanoelectrodes horizontally protrude from the mesh circuit or the walls of microfluidic chambers. Importantly, these electrodes can be fabricated with sizes that smaller than the diameter of a single cell [16]. This configuration exploits the electrodes with high-aspect ratios [53,55,56] and enables recording of action potentials from only a few muscle cells and local field potentials in millimeter-scale animals [57].

In many of these cases, however, the mechanical rigidity of the devices requires that the animals are pressed and immobilized against the nanoprobes or constrained inside the microchannels during electrophysiological recordings, with associated severe impact on normal movements and activities. For more meaningful behavioral research, MEA systems with little to no physical or geometric constraints must be developed.

### Microwires

Electrophysiology with individual or bundled microwires is a common approach for recording in millimeter-scale small animals. To examine odor coding in the *Drosophila* antenna, de Bruyne et al. [58] used a tungsten wire with an electrolytically sharpened tip with diameter of ~1 μm for extracellular recordings. They captured action potentials with different odor stimulations and presented an analysis of individual olfactory receptor neurons (ORNs) as a functional map showing spatial distribution of each ORN class on the antennal surface. To explore temporal patterns of odor coding, Martelli et al. [59] used tungsten wires to investigate physiological aspects of ORN response and physical aspects of odor stimuli and their relationships to diverse responses in *Drosophila* ORNs. Single sensillum recordings amplified, bandpass-filtered (300 Hz to 2 kHz), and digitized at 10 kHz revealed odor-specific stimulus dynamics and ORN dynamics in *Drosophila*. To further investigate mechanisms that define how olfactory neurons process the timing and intensity of odor whiffs, Gorur-Shandilya et al. [60] used microwires to capture the responses of ORNs of fruit flies to these odors.

In addition to extracellular recording for electrophysiology, microwires can be used for programmable electrical stimulation to control simple motions of insects [61–64]. For example, Li et al. [61] reported cyborg honeybees controlled by applying electrical stimulation signals with different duty cycles and frequencies to the unilateral optic lobes. They implanted 2 tungsten wires (50 μm in diameter) for stimulation and one tungsten wire for a ground electrode [61].

### Combining bioelectronics with other monitoring modalities

The integration of different monitoring modalities can overcome the limitations associated with bioelectronics approaches used in isolation. The consequences may lead to findings that enhance our comprehension of the intricate connection between electrophysiological signals and the behavior of small model animals, potentially leading to significant discoveries in the field of biology and beyond. Multiple electrophysiology monitoring technologies, such as calcium imaging and MEAs, yield results that can be compared and contrasted with each other for improved accuracy. For example, Hinz and de Polavieja [65] used idTracker to obtain the trajectory of each animal in a group using a method of image fingerprinting without manual corrections. By obtaining the correct values of quantities derived from trajectory data like velocities or accelerations, they correctly identified individuals in research on the collective behavior of zebrafish. In other work, Das et al. [26] used genome editing, force spectroscopy, and multiscale modeling. They discovered an important mechanical interplay during body movements of *C. elegans*.

For neural signal recording under the influence of multiple variables that must be monitored or controlled at the same time, multiple techniques are essential. An advantage of bioelectronic systems for multimodal research is the integration of experimental modalities, including coordination of simultaneous neural recording, behavioral monitoring, and manipulation of the microenvironment. For example, electrophysiological research can be expanded and enriched with environmental manipulations such as chemical delivery, temperature regulation, and mechanical stimuli [26,39,66–69]. Chemicals can be delivered in real time to the animal during pharyngeal recordings to compare and evaluate some anthelmintic drugs [38,66] or to screen for efficient drugs to treat specific diseases [24]. Moreover, the fluid flow inside the microfluidic chambers can be controlled properly to induce mechanical stimuli with different intensities for spatiotemporal simulation of certain responses [70–72]. Some studies reported the use of such approaches in combination with behavioral measurements or calcium imaging for high-throughput analysis of touch responses in *C. elegans* [73], including research on the role of sensory neuron responses in this process [69]. Testing of changes in sensorimotor responses during sleep represents another example [67]. In addition, the environmental temperature can also be tuned in real time, for example, through the rapid-flow microfluidic methods, for promoting sleep behavior of *C. elegans* [74]. Using this technique, Tzouanas et al. [75] revealed the Hydra behavioral response to temperature stimuli. As a result of this work, they discovered a potential thermally responsive circuit in the Hydra nervous system. Other stimuli based on illumination with light or introduction of food, odorants, and gasses can also be fine-tuned during experiments to study the corresponding behavioral responses [76,77].

## Approaches to Bioelectronics for 3D Electrophysiological Monitoring

This section focuses on 3D flexible bioelectronics for electrophysiological monitoring of soft organisms that are mostly in vitro cellular tissues. Advanced cellular cultures have comparable sizes and mechanics to millimeter-scale animals, and they can benefit from 3D recording capabilities as these cultures (e.g., organoids) tend to detach from the substrate and become freestanding as they mature [18,21]. The organizational scheme categorizes these devices into different forms: ultrathin, stretchable, stress-driven, and biohybrid. Table 3 compares different kinds of 3D flexible electronics technologies for electrophysiological monitoring of soft organisms. Analyses of the underlying challenges and opportunities for applying these methods to millimeter-scale animals appear in the “Next-Generation Bioelectronics for Millimeter-Scale Animals” section.

**Table 3.** Comparisons of existing 3D flexible electronics for electrophysiological monitoring

Technologies	Materials	Fabrication	Stretchability	Bending stiffness/modulus	Impedance at 1 kHz	Tissue	References
Ultrathin electronics, exposure energy difference between the SU-8 bilayers induced architecture, stretchable architecture	Polyimide or SU-8 and a metal conductor	Microfabrication using photolithography and thin-film metal deposition	None–30%	2.9 × 10^−17^–4.5 × 10^−13^ N·m^2^	~10^5^ Ω	Brain organoids and cardiac organoids	[18,21,85,185]
Ultrathin electronics, different modulus distributions	Poly(styrene–ethylene—butylene–styrene) elastomer and liquid metal	Selective laser ablation, stencil print, and thin-film metal deposition	Up to 400%	1.3 MPa (pristine elastomer)	<1 kΩ under 300% strain	The surface of a bullfrog heart	[186]
Intrinsic differential stress-induced architecture	SiO/SiO_2_ and a metal conductor	Microfabrication using photolithography, thin-film metal deposition, and thermal chemical vapor deposition	–	Depending on the thickness	Depending on the choice of conductor	Live cells	[187]
Substrate-free grafting of conducting materials with tissue	Conducting polymer gels	Polymerization triggered by an enzyme in cells	–	Depending on the thickness	<10^5^ Ω	Live zebrafish and leeches	[37]
Residual stress in thin-film metals induced architecture	Polyimide or SU-8, graphene field-effect transistors, and a metal conductor	Microfabrication using photolithography, thin-film metal deposition, and low-pressure chemical vapor deposition	–	Depending on the thickness (<2 μm)	(1.4 ± 7.6) × 10^5^ Ω	Human electrogenic spheroids	[87]

### Ultrathin electronics

**Fig. 2.**
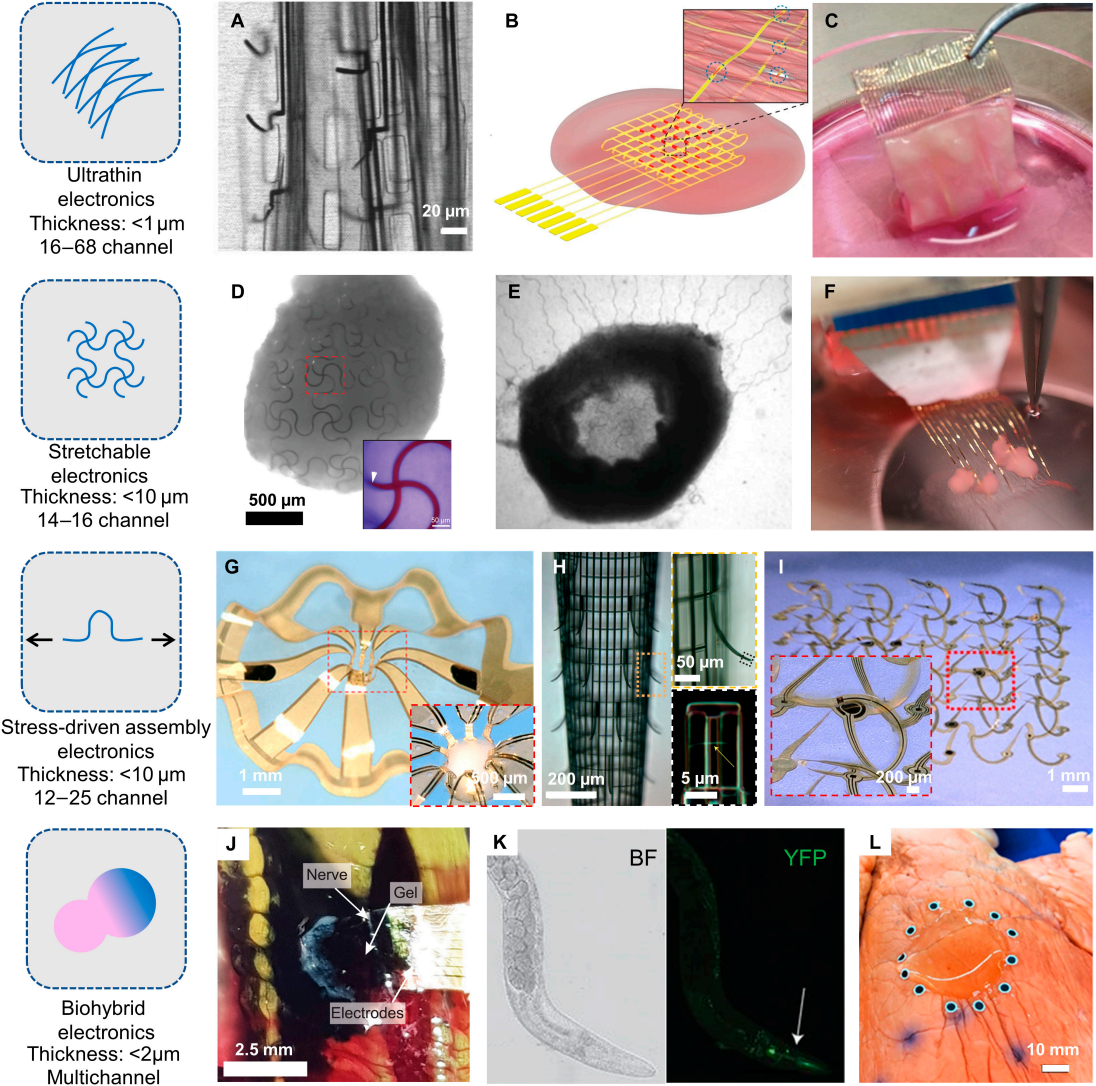
Approaches to 3D bioelectronics for soft organisms. (A) Bright-field images of a 50-μm-thick brain slice with an implanted 100-nm-thick NEAs. Adapted from [17]. (B) Schematic illustration of a nanoelectronic scaffold with cardiac tissue resulting from the cultivation of cardiac cells within the 3D folded scaffold. Adapted from [21]. (C) A photograph of a folded microelectronic cardiac patch after 7 d of cultivation with cardiac cells. Adapted from [81]. (D) Uniform distribution of stretchable mesh nanoelectronics on the bottom of an organoid, with an inset that shows a magnified view of the region highlighted by the red dashed box. Adapted from [18]. (E) Optical phase images of human induced pluripotent stem cell-derived neurons integrated with stretchable mesh nanoelectronics, forming a 3D cyborg brain organoid. Adapted from [85]. (F) Meshed microelectronics integrated with human cortical organoids. Adapted from [86]. (G) Prestrain-induced mesostructure of 3D electronics with 25 electrodes, with an inset that shows a cortical spheroid enclosed in the electronics. Adapted from [88]. (H) A macroporous nanoelectronic brain probe suspended in buffer with a cylindrical shape. Adapted from [92]. (I) Optical images of various 3D electronic scaffolds. Adapted from [84]. (J) In vivo polymerization around the nervous system of medicinal leeches using flexible probes. Adapted from [37]. (K) Bright-field (BF) (left) and fluorescence [yellow fluorescent protein (YFP)] images (right) of *C. elegans* expressing *Pmyo-2::Apex2::mcd8::gfp*-labeled Apex2(+) versus wild-type controls labeled Apex2(–). The arrow indicates green fluorescent protein-labeled pharyngeal muscle and Apex2 expression. Adapted from [97]. (L) A photograph of a 3D printed circular layer of hydrogel on a porcine lung for in situ monitoring of deformation. Adapted from [99].

Electronics that are sufficiently thin and porous can easily wrap onto arbitrary biological surfaces without additional adhesives due to low bending stiffnesses and strong Van der Waal’s interactions at the contacting interfaces. Devices of this type are usually fabricated by photolithography techniques, with micrometer-thick photodefinable polymers as dielectric layers and nanoscale films of gold as conductors. The electrode–tissue interfaces exploit nanowire-based field-effect transistors [78–81] or noble metal electrodes [82–84], specifically designed to collect the low-amplitude signals associated with extracellular electrophysiology. To overcome the mismatch of electrode–tissue interfaces caused by micrometer-thick polymer substrates, Gao et al. [17] developed self-assembled nanofilm electrode arrays (NEAs) that consist of high-density, freestanding gold nanofilm electrodes. These chronically implanted NEAs can form intimate and innervated interfaces with neural tissue, enabling stable 3D neuronal activity recordings over several months. Figure 2A shows the brain slice image with the implanted self-assembled NEAs. Tian et al. [80] reported silicon nanowire field-effect transistors integrated into ultrathin, mesh electronic layouts. The device exploited a porous and freestanding cellular scaffold with a 2D open porosity of 75% and a thickness of 2 μm, which enabled it to roll into a 3D structure that was further hybridized with biomaterials for neurites and cardiomyocyte seeding. Dai et al. [21] reported scalable mapping and regulation of action potential propagation in ultrathin nanoelectronics-innervated cardiomyocytes tissues (Fig. 2B). Feiner et al. [81] reported similar mesh-structured ultrathin electronics with recording electrodes and an on-demand drug-releasing network (Fig. 2C).

These ultrathin electronics are capable of electrophysiological mapping of cellular tissues with 3D spatial resolution and high temporal resolution, with the potential for manipulating cellular behaviors by electrical stimulation. Application to millimeter-scale animals may be possible by improving the limited mechanical stretchability (typically <10%) of these ultrathin electronics to allow accommodation of large movements of ambulating animals.

### Stretchable electronics

Long-term electrophysiological monitoring of millimeter-scale animals requires the devices to endure substantial deformations caused by the growth and movements. Stretchability of bioelectronic systems can be enhanced by incorporating stretchable geometries or intrinsically stretchable materials.

Li et al. [18] reported stretchable mesh nanoelectronics that exploit a stretchable serpentine geometry. The device can seamlessly laminate onto a layer of growing stem cells due to its ultralow thickness (<1 μm) and light weight. As the stem cells differentiated into cardiomyocytes and self-organized into a 3D organoid in ~20 d of cellular culture, the volumetric expansion was up to 3 times compared to the initial cellular state. The nanoelectronics remained functional due to the large biaxial stretchability (~30%) (Fig. 2D). The resulting organoid-electronic hybrid, or “cyborg organoid”, can be used to study the evolution of electrophysiology throughout the entire organogenesis via the 16 embedded microelectrodes from day 0 of cell culture. Similarly, Le Floch et al. [85] reported stretchable nanoelectrode arrays that can integrate with growing brain organoids (Fig. 2E). The seamless and noninvasive coupling of electrodes from day 0 of culture enabled long-term and continuous 3D mapping of neuronal activities as the organoids developed. When coupled with single-cell transcriptomics, the results allow detailed studies of the development of the human brain and neurological disorders using these in vitro organoid models.

As an example of a technology enabled by intrinsically stretchable materials, Li et al. [86] reported stretchable mesh electronics for long-term interfacing with free-floating human cortical organoids (Fig. 2F). The mesh consisted of electrode arrays made of poly(3,4-ethylenedioxythiophene) polystyrene sulfonate-based electrically conductive hydrogel and dielectrics made of elastomeric poly(styrene–ethylene–butylene–styrene), both of which are intrinsically stretchable polymeric materials. Released from the silicon wafer with a water-soluble dextran sacrificial layer, this device can maintain a stable electrochemical impedance in buffer solution with up to 50% cyclic strain. The device established a stable electrical interface with human cortical organoids for more than 3 months. In addition to electrophysiological measurements, factors such as pH and neurotransmitter concentrations were also monitored using electrochemical electrodes.

### Stress-driven assembly electronics

Another approach for realizing 3D geometric matching with biological tissues is by stress-driven self-assembly. Kalmykov et al. [87] reported a 3D self-rolled biosensor array implemented with either graphene field-effect transistors or metal electrodes that can noninvasively envelop a cardiac spheroid. With 12 electrodes arranged in 3D layouts, the device can simultaneously record field potentials and provide information regarding the frequency of beating. Park et al. [88] developed 3D bioelectronics that can fold and gently surround the brain organoid to establish a robust 3D electrode–tissue interface (Fig. 2G). Complex architectures and high-resolution features highlight its design versatility. The neuronal activities of the organoid were recorded by the conformal electrode arrays with up to 25 channels. The spatially distinct, high-resolution field potentials across all the channels highlighted the utility of such a system in investigating long-term and spatially resolved biological events. Furthermore, recent progress in the fabrication of 3D electronics paves the way for the formation of deterministically engineered assembloids. For example, complex 3D mesostructures hosting microelectronic circuits can be mechanically assembled through shape-memory effects of either the underlying elastomer substrate [89] or the freestanding 2D thin-film precursors [90]. These microfabrication technologies led to transparent, compliant 3D mesostructures that mechanically conformed to organoids for precise evaluation of their mechanical characteristics [91].

For brain probes that implant into millimeter-scale animals, the dimensional and mechanical mismatches with the brain tissue limit their stability and signal fidelity. Xie et al. [92] utilized built-in residual stress in thermally evaporated metal films for inducing highly controlled and localized 3D self-assembly of an initially planar mesh microelectronics (Fig. 2H). The probe-like structure after assembly facilitated implantation into the brain and increased electrode–neuron contact during long-term recording.

To precisely control the geometry of 3D electronics, Wang et al. [84] developed a strategy depending on geometric transformation of 2D precursors by compressive buckling (Fig. 2I). The device can achieve scalable distribution of multiple functional components for monitoring and regulation of tissue cultures, with multimodal sensors for pressure, electrophysiology, and temperature.

### Biohybrid electronics

An additional type of bioelectronics comprises electrically active biomaterials formed within the living organisms, also referred to as biofabrication or in situ polymerization [93–96]. These emerging techniques show promise in further improving biocompatibility and stability of the electrode–tissue interface.

Strakosas et al. [37] reported the formation of electrodes in situ by substrate-free grafting of conducting materials within living zebrafish and leeches. Conducting polymer gels formed by triggering enzymatic polymerization of organic precursors within an injectable gel. This approach can be used to target specific biological substructures for fully integrated, in situ fabricated 3D electronics within the nervous system. To form the electrodes, gels were injected into the brains of zebrafish, and the animals were left for several hours to allow full polymerization of the gel. MEAs were then placed on top of the brain slices for electrophysiological recordings in regions of dark spots (Fig. 2J). In another work, Liu et al. [97] genetically engineered living neurons to guide chemical synthesis of electrically functional (conductive or insulating) polymers, realizing remodeling of membrane properties and modulated cell-type-specific behaviors in freely moving animals (Fig. 2K). They designed a single-enzyme-facilitated polymerization using chemically modified monomers, for which polymerization was triggered by an enzyme that expressed in specific cells. Small-molecule conductive polymer precursors were delivered into intact animal tissues through diffusion.

These biohybrid electronic systems have interesting performance attributes but face challenges in the manufacturing processes especially during establishing electrical connections with other electronic components. Despite these challenges, the superior biocompatibility and long-term stability of biohybrid electronics suggest potential for advancing the fields of brain-machine interfaces and cyborg organs.

As a final example, a combination of 3D printing, microfabrication, and tissue engineering allow formation of functional electronic structures directly on top of organs. Zhu et al. [98] reported a hybrid manufacturing technique that combines 3D printing of wires with automatic pick and placing of surface-mounted electronic components. Operable electronic devices can be maintained in a free-moving human hand. Cell-laden hydrogels printed on live mice use the same method. They further developed a 3D printing system that can directly print on the surface of a breathing lung (Fig. 2L) by combining offline shape learning with online shape tracking [99]. An electrical impedance tomography sensor integrated with ionic hydrogel ink yielded a soft wearable sensor for in situ mapping of 2D volumetric strain with high spatiotemporal resolution. This in situ adaptive 3D printing technology provides a valuable avenue for fabricating electronic devices directly onto biological tissues, potentially facilitating integration with millimeter-scale animals.

## Electrophysiological Studies of Behaviors in Millimeter-Scale Animals

Although freestanding 3D bioelectronics have significantly promoted electrophysiological studies, research progress on millimeter-scale animals utilizing advanced flexible electronic technologies is still in the early stages. A key requirement is to simultaneously record neural activities and the corresponding behavioral responses, whether for medical applications like studies of disease progressions and drug screening [100–102] or for examining the neural underpinnings of certain behaviors [57,103–105]. Existing technologies for electrophysical monitoring of millimeter-scale animals are limited, thereby hindering studies on their individual and social behaviors. In contrast, larger model animals like rats can tolerate larger and heavier electronic implants with more sophisticated functions [106]. This section discusses behavioral studies on millimeter-scale animals categorized into individual and social behaviors.

### Individual behavior

**Fig. 3.**
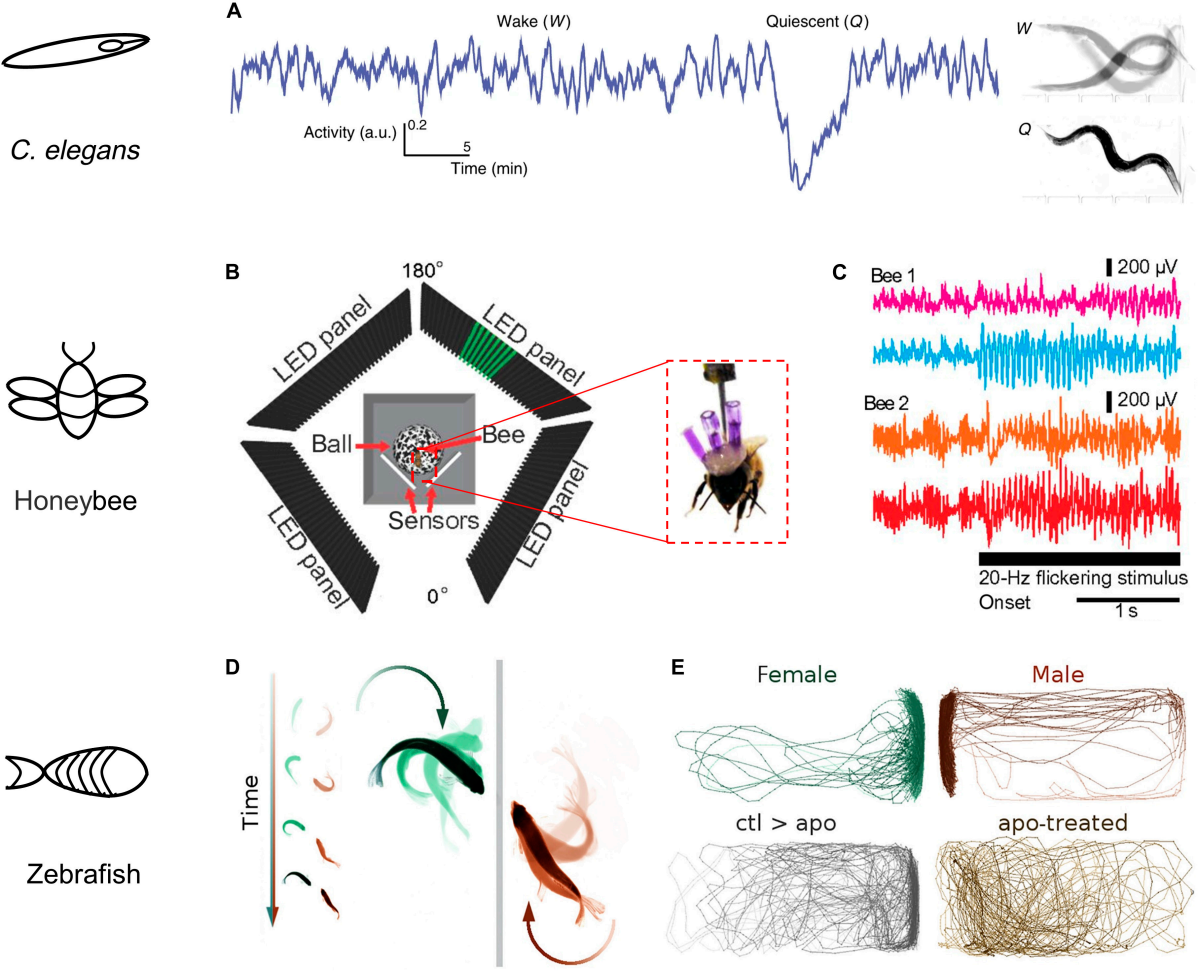
Electrophysiological studies of behaviors in millimeter-scale model animals. (A) The 1-h activity trace of a swimming worm is shown in the left with quiescence hallmarked by a clear drop. The 1-s overlap of frames in the right shows the animal swimming during the wake period and immobile during quiescence. Adapted from [74]. (B) Illustration of the setup for inducing behaviors via visual attention of a semifree honeybee. Adapted from [110]. (C) Neural activity recordings of visual attention in behaving honeybees. Adapted from [110]. (D) Social behavior of a pair of zebrafish in isolated tanks over time. Adapted from [125]. (E) Representative traces for a male and female ABxTU dyad and a control (ABxTU) animal (ctl > apo) paired with an impaired apomorphine-treated zebrafish (apo-treated; apo). Adapted from [125]. a.u., arbitrary units; LED, light-emitting diode.

Millimeter-scale animals are increasingly valuable subjects for neurobehavioral studies due to their wide availability as human disease models, ease of cultivation, and short life cycles [105,107]. For example, Gonzales et al. [35] reported a device with nanoscale-spear-like electrode arrays for scalable electrophysiology of *C. elegans*. This device relies on immobilized *C. elegans* within microfluidic channels, for extended recording with nanospear electrodes embedded in the channels firmly pierced into the animal. By utilizing this device, they established the first scalable electrophysiological phenotypes for amyotrophic lateral sclerosis and Parkinson’s disease for *C. elegans* models. They also evaluated a partial rescue of the Parkinson’s phenotype through drug treatment [35]. A different study reported a scalable platform for tracking animal movements and performing whole-brain imaging [74]. This system established a model for studying how animals process multiple sensory pathways to regulate behavioral states (Fig. 3A). When establishing correlations between individual animal behaviors and electrophysiology, algorithms for automated experimentation and analyses are important [26,108,109].

While growing numbers of bioelectronics technologies can continuously record electrophysiological signals from the central nervous system of millimeter-scale animals, it is necessary to consider how to evoke and describe the representative behaviors. In one example, Paulk et al. [110] discovered the selective responses of honeybees to salient visual stimuli as an attention indicator. They conducted a closed-loop experiment with tethered, walking bees controlling virtual objects in a virtual reality arena (Fig. 3B). They used glass electrode tips filled with saline and fine wires threaded into the electrodes to record brain activities. Monitoring selectivity in the medulla (an optic ganglion) preceded behavioral selection of a stimulus when bees were presented with competing frequency-tagged visual stimuli (Fig. 3C). The results suggest that modulation of early visual processing centers precedes eventual behavioral choices made by these insects. One noteworthy result from their study was the absence of attention-like effects for steady-state visually evoked potentials in the central brain compared to the inner optic lobes, which differs from previous research results [111–113]. As another example, research finds that mushroom body neurons can respond to olfactory stimuli in certain insects [114]. Recording the activity of large single neurons in the central brain may better reflect the participation of the mushroom bodies and central complex in insect behaviors. Thus, future challenges are to identify the decision-making circuits in the central brain that might govern the population-level selective responses in the optic lobes. Such studies can be aided by long-term, minimally invasive electrophysiological recording techniques.

Apart from recording, electronics can also be utilized to modulate certain activities of millimeter-scale animals by electrical stimulation. Behavioral manipulation methods using the innate mobility and crypticity of millimeter-scale animals, such as locusts and cockroaches, can be applied in reconnaissance and rescue missions that are inaccessible to humans or traditional robots [63,64]. These biohybrid robots require computer algorithms for bionic modeling [115,116] and necessitate an understanding of the relationship between the muscular behaviors and electrophysiology [40]. For example, Ma et al. [63] reported a biohybrid jumping robot that retained the natural jumping ability of a locust. Utilizing flexible electronics, Kakei et al. [62] reported a cyborg cockroach integrated with a body-mounted ultrasoft organic solar cell, enabling wireless recharging and locomotion control. The solar cell provided a power output of 17.2 mW on the curved abdomen of the insect, allowing for wirelessly controlled turn-right locomotion after 30 min of illumination. Dong et al. [117] developed a highly controllable biohybrid robot using genetically engineered *C. elegans* with disabled signal transmission between neurons and muscular cells, while preserving their optical responsiveness. The robot was controlled by a laser stimulation system, which simulated phase differences between body curvature and muscle activation patterns and used real-time visual feedback during crawling to realize closed-loop control.

### Social behavior

Reduced social interactions caused by the recent pandemic have affected mood, sleep, and eating habits, highlighting the influence of social behaviors on physical and mental health [118,119]. Millimeter-scale animals are important models for studying the influence of social factors on a behavior-related neural basis [120–124]. Many such species rely on social stimuli to determine their next actions, presenting numerous opportunities for social behavior investigations. For example, Stednitz et al. [125] identified genetically defined populations of neurons in the forebrains of zebrafish that are key to social orienting (Fig. 3D and E). Xu et al. [126] studied the food-averse migration of *Drosophila melanogaster* after feeding larvae toward food-free habitats before metamorphosis and evaluated their relevant social response. Dombrovski et al. [127] reported the emergence of cooperative feeding groups among fruit fly larvae, a phenomenon reliant on visually guided interlarval coordinated movements and stable group membership through experience. For electrophysiological studies on behavior, Xu et al. [126] used a fly larva model to delineate the neurobiological basis of age-restricted responses to environmental stimuli and provided evidence for a fructose-responsive chemosensory pathway that modulates food-averse migratory and social behaviors. Applying recently developed machine vision algorithms to track individual animals in a group, Hinz and de Polavieja [65] studied the emergence of the interaction rule in zebrafish during development from hatching to the juvenile stage.

Sharing neuroanatomical and molecular features across taxa, millimeter-scale animals are suitable for studying fundamental circuit mechanisms underlying vertebrate social behavior within experimentally manageable scales. Simultaneous recording of neural activities from multiple millimeter-scale model animals in an untethered, ambulating manner is, however, challenging. Technology is available for larger animals such as rats, where the size and weight requirements are relaxed. For example, Yang et al. developed a robust and flexible platform tailored for stimulating difficult-to-reach brain regions during complex behavioral tasks in multiple free-moving animals [106]. Several technical challenges must be overcome to realize wireless communication devices designed for millimeter-scale animals. One intrinsic issue is that the wireless transmission efficiency of radio-frequency waves scales with antenna size [128]. Future efforts are likely to focus on innovative integrated chip designs that conserve both space and power [129] or unusual formats for wireless transfer beyond radio-frequency techniques [130].

## Next-Generation Bioelectronics for Millimeter-Scale Animals

**Fig. 4.**
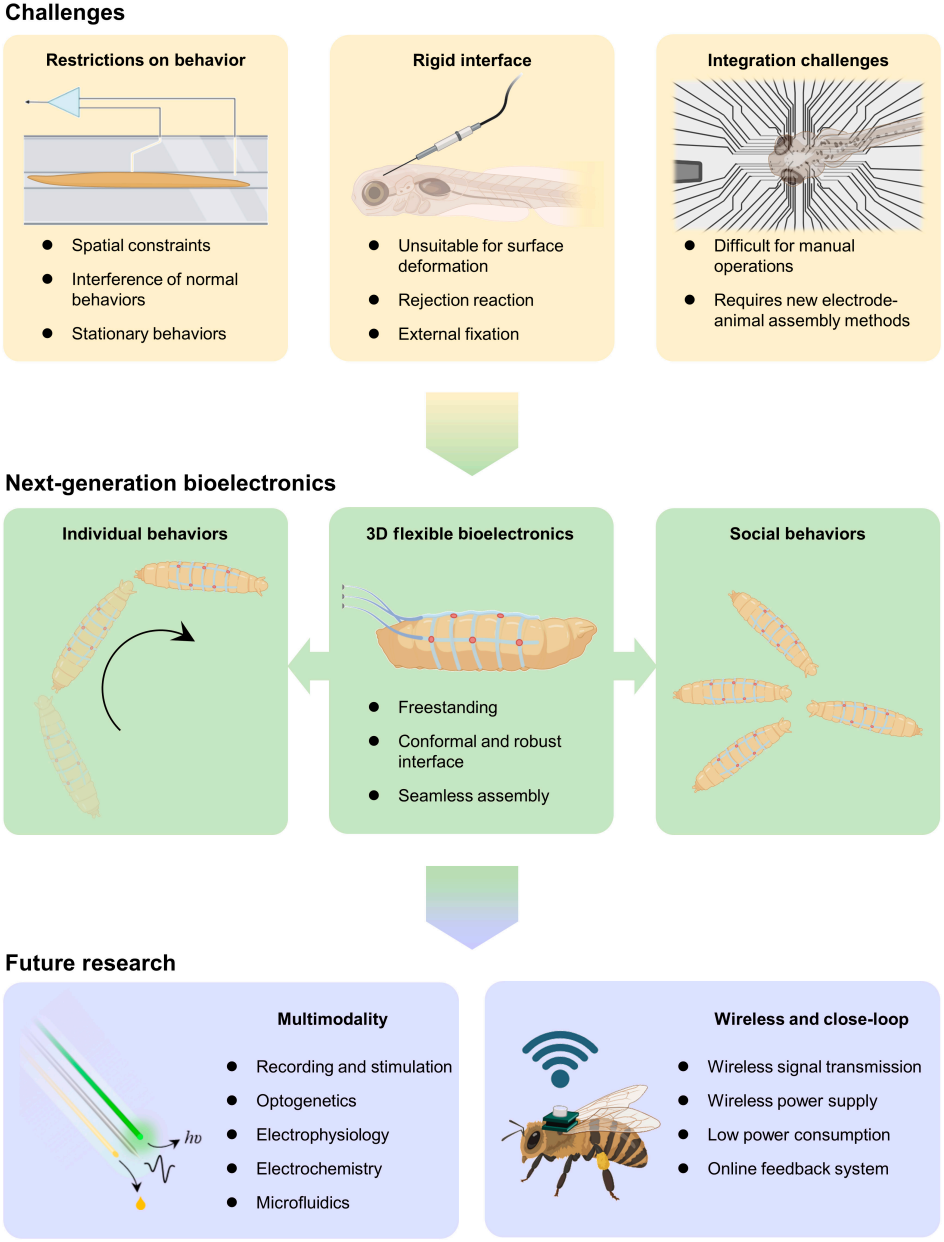
Next-generation bioelectronics for millimeter-scale animals.

For both individual and social behaviors, simultaneous 3D recording of neural signals and small animal behaviors is crucial to decipher the correlation between behavior patterns and neural circuits. Current electrophysiological and imaging techniques applied to small model animals provide insights into the primary input–output functions of neural circuits and the correlations and regulations of behavior. Techniques like patch clamp, MEAs, or calcium imaging impose certain restraints on the animals during signal recording [74,131]. These constraints may not significantly affect neural signals during resting behaviors, but they must be considered for more active, social behaviors that require a high degree of freedom and mutual communications among millimeter-scale animals. Conventional electrical and imaging devices, comprising static and rigid modules, are also incompatible with ambulating animals. The diminutive size of the animals further compounds the challenges associated with manual device integration. These challenges are illustrated in Fig. 4. In addition, different recording modalities present distinct requirements and challenges in neurophysiological investigations. Local field potentials demand high spatial resolution and sensitivity to subtle changes. Extracellular spiking recordings focus on individual neuron action potentials, necessitating precise temporal resolution and signal-to-noise ratios for robust single-unit discrimination. Intracellular spiking recordings require spatial and temporal precision and navigating challenges associated with cell penetration and stability. As discussed above, 3D flexible bioelectronics, characterized by high softness, stretchability, and small thickness, offer the potential to minimize device-related restraints on millimeter-scale animals through a conformal, long-term stable bioelectronic interface (Fig. 4). These devices for millimeter scale animals will focus on local field potentials and extracellular spiking signals for long-term studies of biological rhythms and pathological models. In addition, the rational design of 3D flexible bioelectronics enables multichannel and high-throughput electrophysiological signal recording for comprehensive behavior pattern analysis.

### Confinement limits

Microfluidic chips are highly convenient tools for investigating millimeter-scale animals, offering a wide range of functions, encompassing the capturing, sorting, and immobilization of these organisms [132–134]. These microfluidic devices facilitate detailed neurobiological explorations of millimeter-scale animals by creating fixed imaging locations or compact electrode–tissue contacts. Several microfluidic systems with integrated electrodes can autonomously capture zebrafish larvae, facilitating stable contacts between brains and surface electrode arrays and enabling noninvasive long-term electrophysiological recording from multiple zebrafish [135,136]. These integrated microfluidic platforms provided examples for drug screening with epileptic zebrafish.

Diverse microfluidic devices designs offer interesting capabilities for neurophysiological research. Candelier et al. [137] used an open-ended microfluidic chip to precisely deliver chemical stimuli to agarose-restrained zebrafish larvae, achieving high spatial and concentration accuracy. They correlated gustatory neuronal responses with motor behavior while incorporating whole-brain calcium imaging. Sy et al. [138] developed an optofluidic system to realize highly controlled chemical delivery with laminar flow constraints, allowing the study of chemosensory behaviors of zebrafish larvae. Furthermore, Gonzales et al. [35] established a scalable electrophysiology platform using a microfluidic device integrated with suspended electrodes array. This platform provided precise confinement for mollusks within the microfluidic device, facilitating the exploration of muscle electrophysiological behaviors at the single-cell level in organisms like *C. elegans* and Hydra [35].

While these studies provide valuable insights into neural behaviors, electrophysiological and imaging devices unavoidably impose spatial limitations on millimeter-scale animals [23,74,131]. Next-generation bioelectronics for millimeter-scale animals must address the challenges produced by these spatial confinements, allowing the development of freestanding electrode–tissue interfaces for the study of instinctive behaviors. 3D flexible bioelectronics, as promising candidates, have great potential in minimizing the constraints between electrodes and soft tissues. For instance, a mesh-like structure with a highly porous backbone and materials with high extensibility and low mechanical strength can substantially reduce the electrode weight and rigidity. Accordingly, the unrestrained encapsulation of electrophysiological probes can be further translated into the design and fabrication of “wearable devices” for millimeter-scale animals.

### Robust electrode–tissue contact

Conventional electrophysiological probes made of rigid materials, such as glass, metals, and inorganic semiconductors, are widely used in electrophysiological recording and stimulation of millimeter-scale animals. For example, Yan et al. [139] used whole-cell patch-clamp recording to investigate how body wall muscles in *C. elegans* respond to mechanical stimuli. They found that muscle cells convert mechanical energy into ionic currents independently of synaptic communication, demonstrating mechanosensation through a nonselective cation channel. Shindou et al. [140] also utilized this method to study the active propagation of dendritic electrical signals in *C. elegans*. By inducing depolarization in a major gustatory sensory neuron through sodium chloride stimulation, they activated sensory neurons and conducted mutant experiments that highlighted the critical role of EGL-19, the α1 subunit of L-type voltage-gated Ca^2+^ channels, in regenerative depolarization of sensory neurons. Rees et al. [141] design a quartz-based solid carbon nanopipette electrode with a 250-nm-diameter tip to measure dopamine levels in *Drosophila* with high spatial resolution. The sharp conical shape of the electrodes enabled implantation into dopaminergic centers of the *Drosophila* brain. In addition, the miniaturization and integration of the electrode probes is possible using complementary metal oxide semiconductor technology. For instance, Neuropixel 1.0 and 2.0 provide 960 and 5,120 recording sites, respectively, on a 10-mm-long electrode stem, enabling high-throughput recording of isolated action potentials from hundreds of neurons [142,143]. Abbott et al. [144] integrated 4,096 microdevices onto a silicon-based chip to create a nanoelectrode array capable of simultaneously recording excitatory and inhibitory synaptic connections from over 1,700 neurons [144].

Despite their high performance, electrode probes made from static and rigid materials face challenges when integrated with dynamic living animals. The high rigidity creates difficulties in conforming to the irregular and diverse surfaces of millimeter-scale animals. The invasive processes for transplantation can also trigger foreign-body reactions. Furthermore, maintaining a stable interface often requires external fixation to ensure close contact between the electrode and the biological tissue. Consequently, the development of conformal, homologous, and stable interfaces is crucial for the advancement of next-generation bioelectrodes. With variable topology and adjustable mechanical properties, 3D flexible electrodes not only adapt to the complex spatial distribution [80] but also accommodate the body deformations caused by the movements of millimeter-scale animals, therefore creating robust animal-probe interfaces for long-term study.

### Challenges for biointegration

As previously discussed, the 3D distribution of flexible MEAs can be achieved through topological structural design [18] and material engineering [145]. The primary challenge in establishing freestanding and stable bioelectronic interfaces lies in the application of 3D flexible electrodes onto the millimeter-scale animals. Surgical implantation remains the most direct approach for animals slightly larger in size, such as mice and adult zebrafish. For instance, a parylene-based flexible MEA surgically implanted into the heart and abdomen of adult zebrafish can record electrocardiograph (ECG) for studies of cardiac regeneration after laser damage [146]. As animal size decreases, however, the complexity of surgical procedures increases to an extent that they become difficult or impossible to perform. Wearable devices specifically designed for millimeter-scale animals offer an attractive solution for integration. Zhang et al. [147] developed an ultrasoft silicone-integrated jacket with a waterproof MEA sensor for ECG recording in zebrafish. This jacket featured a zipper construction with multiple teeth to accommodate different fish sizes and a padding component on the back to relieve the dorsal pressure. A stretchable microelectromechanical system cable with a snake-like structure allowed the zebrafish to swim freely. This flexible waterproof device facilitated chronic nonsedated monitoring of zebrafish ECG signals and the study of circadian rhythms in their activity. Further advances include the construction of polydimethylsiloxane miniature fish tanks to realize simultaneous detection of multiple free-moving fish [148]. Metallo et al. [149] applied a parylene-based MEA to record electromyography (EMG) signals of the tobacco hornworm *Manduca sexta*. Through the optimization of geometric parameters, they improved the spatial resolution and the compactness between EMG electrodes and muscle fibers. Both in vivo and in vitro EMG signals of the caterpillars were then monitored and recorded.

For animals with sizes in the millimeter-scale regime, such as *C. elegans* and *Drosophila* larvae, manual operations are nearly impossible. Therefore, to realize assemblies of electrodes and animals, it may help to consider a combination of material and engineering innovations, for example, strain-driven self-assembling flexible electrodes [92], 3D shape-shifting structures [89], assistance from catheter [15] or shape memory polymers [150], and bioelectronics based on biohybrid conducting polymers [37].

## Conclusion and Perspective

Millimeter-scale animals serve as powerful models in the study of neurobiology [23]. They provide living and comprehensive systems for studies of the transition of signals in neural circuits [151–153], genetic manipulations of the nervous system [154,155], and drug screening and treatment of neurological diseases [66,156]. In addition, these models can be correlated with clear behavioral patterns for the study of neuroethology [157]. 3D flexible electronics hold great potential for interfacing with these millimeter-scale animals. The controllable mechanical properties and multidimensional designs can overcome the geometric limits associated with current static and rigid electrode interfaces. The results allow formation of compact conformal electrode–tissue interfaces to improve the robustness and duration of recording. An important research direction is in the topological designs and materials engineering for noninvasive, surgery-free device integration with ambulating millimeter-scale animals. Self-assembling electrodes with 3D shape-shifting structures [158] or shape memory polymers [150] offer some practical ideas. Biohybrid conducting materials also demonstrate the possibility for seamlessly integrating endogenous electronics within organisms [37]. The utilization of diverse fabrication methods constitutes an enabling scheme for the assembly of nanoscale circuits and sensors on 3D free-form surfaces. Examples include direct free-form laser processing [159], adaptive 3D printing with conformal algorithms [98,99], and light-induced mass transfer [160].

In addition, 3D flexible electrodes offer new possibilities for developing diverse, integrated collections of electronic sensors for millimeter-scale animals. Combining optogenetics, electrochemistry, and electrophysiology, multimodal probes can simultaneously record and modulate specific types of neurons in specific brain regions [161]. Despite the high degree of suitability of optogenetics for studies of millimeter-scale animals, conventional optogenetic modules are limited in spatial resolution by the size of the interfaces. Stretchable, ultrathin microscale light-emitting diode probes provide the potential for conformal biointerfaces with high spatial accuracy [162–175]. The integration of the multifunctional modules, such as electrophysiological electrodes, flexible light-emitting diodes, stimulation electrodes, and drug reservoirs can be realized in the 3D flexible electrodes with precise positioning, covering multiple regions and depths of the nervous system [4]. In addition, decoupling of multimodal sensors is another challenge for engineering of multifunctional devices. Different types of sensing mechanisms or electrical signaling techniques and integration of multiple sensors with distinct interfaces provide attractive strategies for signal decoupling [176].

Future research topics also include further regulation and combination of electrophysiological recording with other functional systems, such as external physical stimuli and microfluidics with neurotransmitters or drugs that introduce environmental cues [74]. The integration of these real-time signal sensing, processing, and executive functions with wireless modules shows great promise for closed-loop study on the neural circuits of millimeter-scale animals, providing more powerful tools for neurobiology and for the diagnosis and treatment of neurological diseases (Fig. 4) [177]. The primary challenge lies in the miniaturization of wireless devices to meet inherent size constraints of these animals. Functional requirements must be satisfied with small-scale components in dense architectures. Power efficiency is a paramount consideration, given the dominating sizes and weights of batteries. Preferred designs eliminate the need for batteries entirely, using schemes based on body-generated power or wirelessly delivered power from an external source [168,177]. A critical consideration for flying insects, such as bees or *Drosophila*, is in reliable, long-range wireless links that do not impede natural behaviors. Compact data transmission and power management circuits, such as those available in Bluetooth low-energy system on a chip technology, can not only conserve power but also help optimize the signal-to-noise ratio [178,179]. A balance between real-time data transmission and efficient data storage, with compression algorithms, can be facilitated through event-based triggering and local data processing mechanisms.

Besides, imaging methods provide a commendable spatial resolution, allowing for the comprehensive examination of entire brain regions and the meticulous tracking of individual neuronal activities in animals with a transparent body. These imaging methods can be further combined with transparent freestanding MEAs to gather multimodal spatiotemporal information [180,181], enhancing the depth and breadth of neurophysiological investigations. State-of-the-art electrical recording and biointerface techniques designed for lager animals like rodents [15,182,183] have not been applied to millimeter-scale animals. Further reduction in the device dimension, increase in the electrode density, utilization of more stretchable materials, and, most importantly, optimization in the implantation procedures will facilitate the application of these existing techniques to millimeter-scale animals. Through multidisciplinary research efforts, we anticipate that these multifunctional bioelectronics for small model organisms will be widely accessible in neuroscience applications in the years to come.
